# Epigenetic modulators as therapeutic targets in prostate cancer

**DOI:** 10.1186/s13148-016-0264-8

**Published:** 2016-09-15

**Authors:** Inês Graça, Eva Pereira-Silva, Rui Henrique, Graham Packham, Simon J. Crabb, Carmen Jerónimo

**Affiliations:** 1 Cancer Biology and Epigenetics Group—Research Center (CI-IPOP), Portuguese Oncology Institute of Porto (IPO-Porto), Research Center-LAB 3, F Bdg, 1st floor, Rua Dr. António Bernardino de Almeida, 4200-072 Porto, Portugal; 2 School of Allied Health Sciences (ESTSP), Polytechnic of Porto, Porto, Portugal; 3 Department of Pathology, Portuguese Oncology Institute of Porto (IPO Porto), Rua Dr. António Bernardino de Almeida, 4200-072 Porto, Portugal; 4 Department of Pathology and Molecular Immunology, Institute of Biomedical Sciences Abel Salazar—University of Porto (ICBAS-UP), Porto, Portugal; 5 Cancer Research UK Centre, Cancer Sciences, The Somers Cancer Research Building, University of Southampton Faculty of Medicine, Southampton General Hospital, Southampton, S016 6YD UK

**Keywords:** Prostate cancer, DNMTi, Histone modulators

## Abstract

Prostate cancer is one of the most common non-cutaneous malignancies among men worldwide. Epigenetic aberrations, including changes in DNA methylation patterns and/or histone modifications, are key drivers of prostate carcinogenesis. These epigenetic defects might be due to deregulated function and/or expression of the epigenetic machinery, affecting the expression of several important genes. Remarkably, epigenetic modifications are reversible and numerous compounds that target the epigenetic enzymes and regulatory proteins were reported to be effective in cancer growth control. In fact, some of these drugs are already being tested in clinical trials. This review discusses the most important epigenetic alterations in prostate cancer, highlighting the role of epigenetic modulating compounds in pre-clinical and clinical trials as potential therapeutic agents for prostate cancer management.

## Background

### Prostate cancer

Prostate cancer (PCa) is one of the most common malignancies worldwide and a leading cause of cancer-related morbidity and mortality [1]. When diagnosed at early stages, it is potentially curable by radical prostatectomy or radiotherapy [2]. Furthermore, in many men, the disease is in fact indolent raising an important unmet need to better understand the biology of those prostate cancers that will never require exposure to treatment. However, for PCa that recurs after failure of primary surgery/radiotherapy or hormone-naive metastatic disease, androgen deprivation therapy (ADT), combined with docetaxel chemotherapy in suitably fit patients, is the mainstay of treatment [3–5]. Gonadotropin-releasing hormone (GnRH) agonists or antagonists, initially combined with anti-androgens (e.g., bicalutamide), are used to lower androgen levels, leading to tumor remission and a decline in serum prostate-specific antigen (PSA). Although nearly all patients respond to ADT, for patients with metastatic cancer progression to a lethal stage of the disease, termed castration-resistant prostate cancer (CRPC), occurs in virtually all patients after a median of 11 months [6, 7]. Despite previously being termed “hormone refractory” in fact, CRPC normally remains, at least initially, critically dependent on androgen receptor (AR) signaling. The mechanisms underlying castration resistance relating to the AR itself include receptor amplification, activating mutations, constitutively active truncating splice variants, phosphorylation, and methylation. Persistent transcriptional AR activity can also be mediated by altered responsiveness to, or increased expression of, alternative ligands including progesterone and corticosteroids or by adrenal production of androgens that is not responsive to GnRH agonists/antagonists, as well as intraprostatic testosterone and dihydrotestosterone (DHT) synthesis [8]. Finally, components of the activated AR complex, including epigenetic mediators as described in this review, may be overexpressed (co-activators) or repressed (co-repressors) and other signaling pathways may also be activated, for example, including the MAPK, PI3K/Akt, and Wnt pathways [9, 10]. For metastatic CRPC (mCRPC), treatment with next-generation hormonal therapies, such as the CYP17A1 inhibitor abiraterone which depletes androgen synthesis pathway precursors or the AR antagonist enzalutamide, is an option; however, acquired resistance inevitably arises (within 1–2 years in the pre-docetaxel setting) [11, 12]. It is becoming increasingly clear that this clinical phenotype is commonly characterized by therapeutic cross resistance, at least between available hormonal therapies, making sequential use of limited benefit, and that current treatment options drive the emergence of treatment-resistant clonally convergent subpopulations [13, 14]. For mCRPC, other agents that have an established survival benefit include chemotherapy with either docetaxel or cabazitaxel combined with prednisone, the radiopharmaceutical radium-223, and the autologous cellular immunotherapy sipuleucel T [15–19]. Unfortunately, none of these agents are curative and the median survival from the point of transition to mCRPC is 2–3 years [7], strengthening the urgent need for investigation of new therapeutic approaches.

### DNA methylation and histone modifications in prostate cancer

**Fig. 1:**
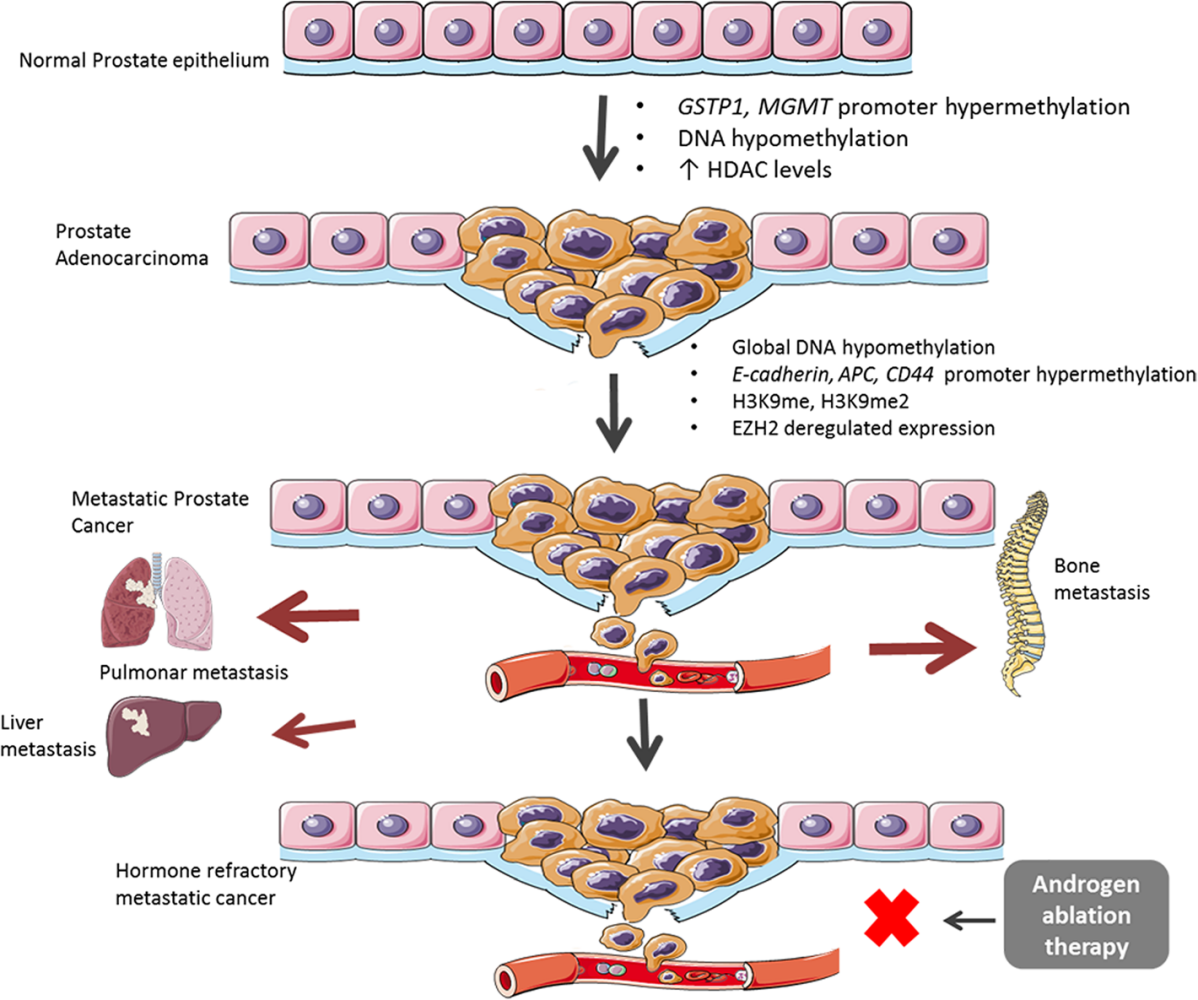
Epigenetic alterations involved in PCa development and progression. Several epigenetic aberrations, as silencing of tumor suppressor genes by promoter hypermethylation, aberrant expression of histone modulating proteins, and DNA hypomethylation contribute not only to PCa onset but also to its progression to advanced and castration-resistant cancer

PCa is a complex and heterogeneous disease that arises from both genetic and epigenetic alterations [20]. Concerning epigenetic modifications, DNA methylation is the best well-studied epigenetic alteration [21]. It consists of the addition of a methyl group by DNA methyltransferases (DNMTs): DNMT1, DNMT3A, and DNMT3B, donated by *S*-adenosylmethionine (SAM), to cytosine residues within CpG dinucleotides. Whereas DNMT1 ensures the maintenance of tissue-specific methylation patterns over cellular replication, DNMT3A and DNMT3B are involved in the maintenance and *de novo* methylation of DNA strands [22, 23]. Aberrant alterations of the methylation patterns are common features of PCa development and progression (Fig. 1). Global DNA hypomethylation increases as the disease progresses, with a lower overall content of 5-methylcytosine (m^5^C) found in metastatic tissues [24], promoting chromosome instability, activation of retrotransposons, and aberrant gene expression. Loss of imprinting of *IGF2* (with consequent biallelic expression) was found in cancerous as well as in associated histologically normal peripheral zone prostatic tissue, which indicates that it might predispose the development of carcinogenesis over a long latency period [25]. Promoter hypomethylation may result in the activation of proto-oncogenes, although this is a relatively underexplored event. One example is urokinase plasminogen activator (*PLAU*), a gene involved in tumor invasion and metastasis and whose expression has been associated with CRPC [26]. Heparanase, an endo-β-D-glucuronidase, is also highly expressed in PCa, especially in metastatic lesions, but not in prostatic intraepithelial neoplasia (PIN) [27]. Conversely, DNA hypermethylation at specific gene loci is a key molecular hallmark of PCa. In fact, this is one of the first aberrations, seen as early as in pre-invasive lesions, such as PIN, and persisting throughout disease progression [28]. Tumor suppressor genes silenced by promotor hypermethylation in PCa are involved in important cellular pathways, including cell cycle control, apoptosis, DNA damage repair or hormonal response. Thus far, more than 100 genes have been shown to be inactivated by promoter hypermethylation in PCa. Remarkably, glutathione S-transferase pi 1 (*GSTP1*), a gene involved in DNA repair, is hypermethylated in more than 90 % of PCa cases, as well as in over 50 % of PCa precursor lesions, suggesting this as an early event in prostate carcinogenesis [29–32]. Methylation of Ras association domain family protein 1, isoform A (*RASSF1A*) promoter was strongly correlated with an increased risk of PCa recurrence, aggressiveness, and tumor progression [33, 34]. Progression to CRPC was also linked with AR silencing by hypermethylation [35]. In fact, AR hypermethylation was described in about 30 % of CRPC [36]. Several other genes were described as frequently hypermethylated in morphologically normal prostate tissue and in PIN (e.g., ATP binding cassette subfamily B member 1 (*ABCB1*), adenomatous polyposis coli (*APC*), cyclin D2 (*CCND2*), *O*-6-methylguanine-DNA methyltransferase (*MGMT*)*,* retinoic acid receptor beta 2 (*RARβ2*), *RASSF1A*, prostaglandin-endoperoxide synthase 2 (*PTGS2*)) further implicating DNA hypermethylation in PCa initiation [37–39].

**Fig. 2:**
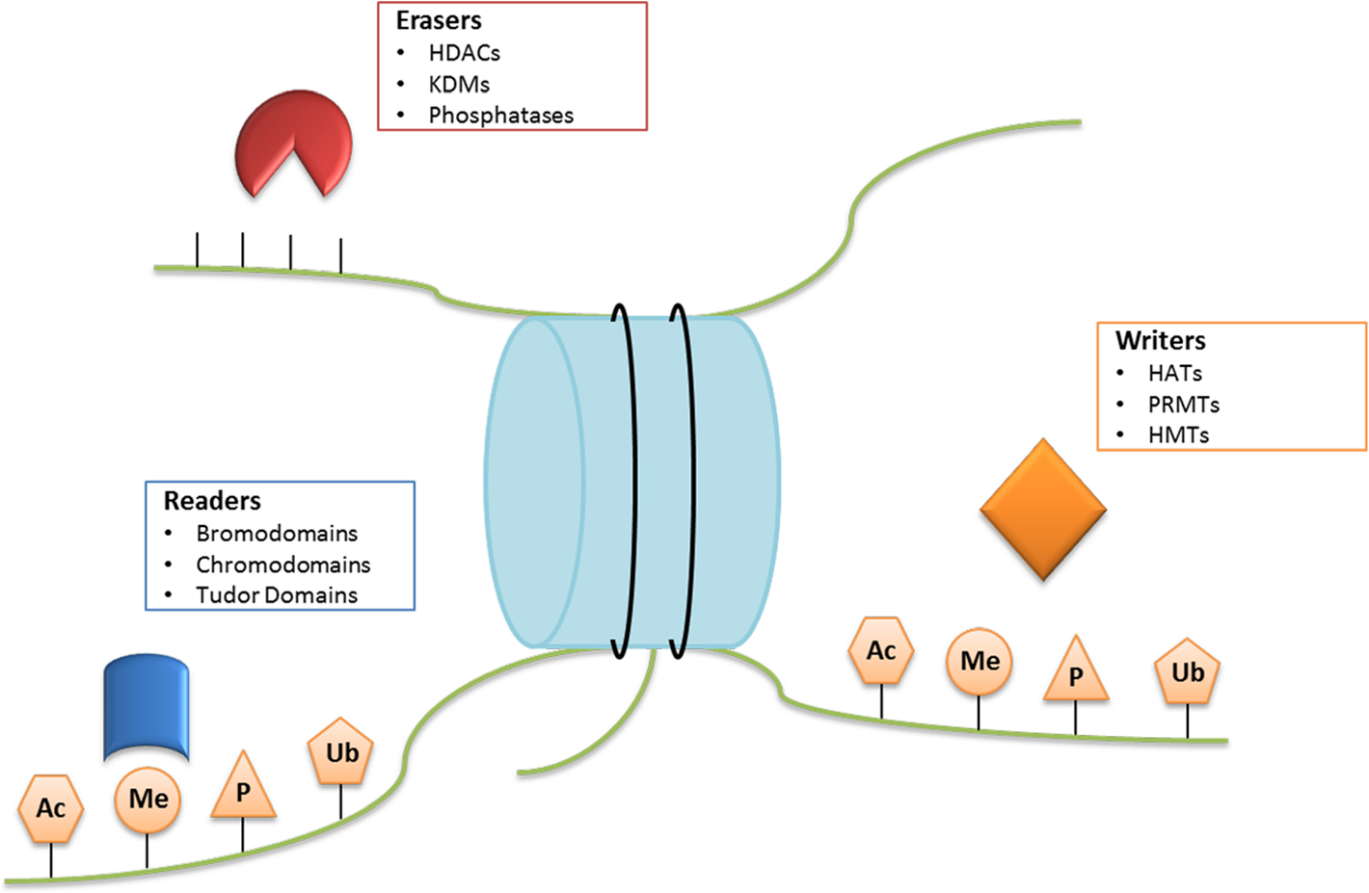
Writers, Erasers, and Readers. Epigenetic Writers (HATs, HDMs, and PRMTs) are responsible to establish epigenetic marks on amino acid residues of histone tails. Epigenetic Erasers (HDACs, KDMs and phosphatases) participate on the removal of the epigenetic marks. Epigenetic Readers (bromodomain, chromodomain and Tudor domain proteins) recognize and bind to a specific epigenetically modified mark

In addition to DNA methylation, histone modifications were also implicated in prostate carcinogenesis (Fig. 1). The N-terminal tails of histones may undergo a variety of post-translational covalent modifications, which are catalyzed by various histone-modifying enzymes (Fig. 2). At least 16 different post-translational modifications (PTMs) have been reported, including acetylation, methylation, phosphorylation, ubiquitination, and glycosylation [40]. These changes constitute the “histone code” which acts as a layer of epigenetic regulation of gene expression affecting chromatin structure and remodeling [41]. In general, acetylation enables transcriptional activity and is catalyzed by histone acetyltransferases (HAT). Conversely, histone deacetylases (HDACs) remove acetyl groups leading to condensed and repressive chromatin. In PCa, HDAC 1, 2, and 3 are strongly expressed, especially in CRPC [42, 43]. Moreover, HDAC1 and HDAC2 were found to be highly expressed in PCa with high Gleason score and might be correlated with increased proliferative capacity [43] but only HDAC2 expression has been associated with shorter PCa patient relapse-free survival time after radical prostatectomy. Additionally, HATs and HDACs may change the acetylation status of non-histone proteins, such as AR [44]. Indeed, AR co-activators and co-repressors influence transcriptional activity by regulating AR itself or its responsive genes, via their respective HAT or HDAC activities. Acetylation of coactivators enhances the transcriptional activity of AR facilitating its binding to target DNA sequences. Contrarily, AR activity is abrogated by HDAC1, HDAC2, and sirtuin 1 (SIRT1) [45]. SIRT1 was shown to be downregulated in PCa, compared to normal prostatic tissue, leading to H2A.Z overexpression and consequent upregulation of v-myc avian myelocytomatosis viral oncogene homolog (*MYC*) and other oncogenes [46].

Histone methylation may be associated with transcriptional activation or repression, depending on the amino acid residue and the number of methyl groups added. Specifically, methylation of lysines 4, 36, and 79 of histone 3 (H3K4me3, H3K36me, and H3K79me) are marks of active transcription, whereas methylation of lysines 9 and 27 of histone 3 (H3K9 and H3K27) results in silent chromatin state [40, 47]. In PCa, H3K4me, H3K9me2, H3K9me3, and acetylation of H3 and H4 were shown to be reduced in comparison with non-malignant tissue. It was also demonstrated that CRPC patients displayed increased levels of H3K4me, H3K4me2, and H3K4me3 [48]. In fact, high levels of lysine-specific demethylase 1A (KDM1A) was correlated with increased risk for disease relapse [49] and AR function [50]. The histone methyltransferase (HMT) polycomb protein enhancer of zeste homolog 2 (EZH2) is by far the most studied epigenetic enzyme in PCa. This enzyme, responsible for H3K27 trimethylation, was found to be overexpressed in PCa, particularly in mCRPC [51] and was associated with promoter hypermethylation and repression of some tumor suppressor genes, suggesting its involvement in PCa progression [51, 52]. Interestingly, in CRPC, the oncogenic role of EZH2 was independent of its polycomb transcriptional repressor activity, functioning as a co-activator of several transcription factors such as AR [53]. Thereby, epigenetic deregulation of co-activators may contribute to failure of androgen deprivation therapy in PCa patients. Lysine-specific demethylase 1 (LSD1) is another enzyme involved in prostate carcinogenesis. It acts both as co-activator and co-repressor of transcription by targeting H3K4 or H3K9, respectively [49, 54, 55]. In fact, LSD1 was found to form a complex with AR, stimulating its activity. Moreover, increased levels of LSD1 were associated with aggressive CRPC and high risk of disease relapse [49, 55].

Several other histone-modifying enzymes, like JHDM2A, JMJD2C, SET9, and SMYD3 have already been shown to play a role in prostate carcinogenesis [50, 56–59]. Moreover, in addition to changes in chromatin modifier enzymes, some histone modifying patterns, like H3K18Ac, H3K4me2, and H3K4me1 were also associated with increased risk for PCa recurrence [48, 60].

### Evidence acquisition

We searched PubMed for publications on PCa and epigenetic therapy using the keywords: prostate cancer, DNA methylation, histone modifications, epigenetic drugs, DNMT inhibitors, HDAC inhibitors, histone modulators, HAT inhibitors, histone demethylase (HDM) inhibitors, and every drug mentioned on the manuscript, on January 15, 2016. Only articles written in English were retrieved. Original reports were selected based on the detail of analysis, mechanistic support of data, novelty, and potential clinical usefulness of the findings. A total of 283 papers were included in this review.

## Epigenetic silencing as a therapeutic target in prostate cancer

**Fig. 3:**
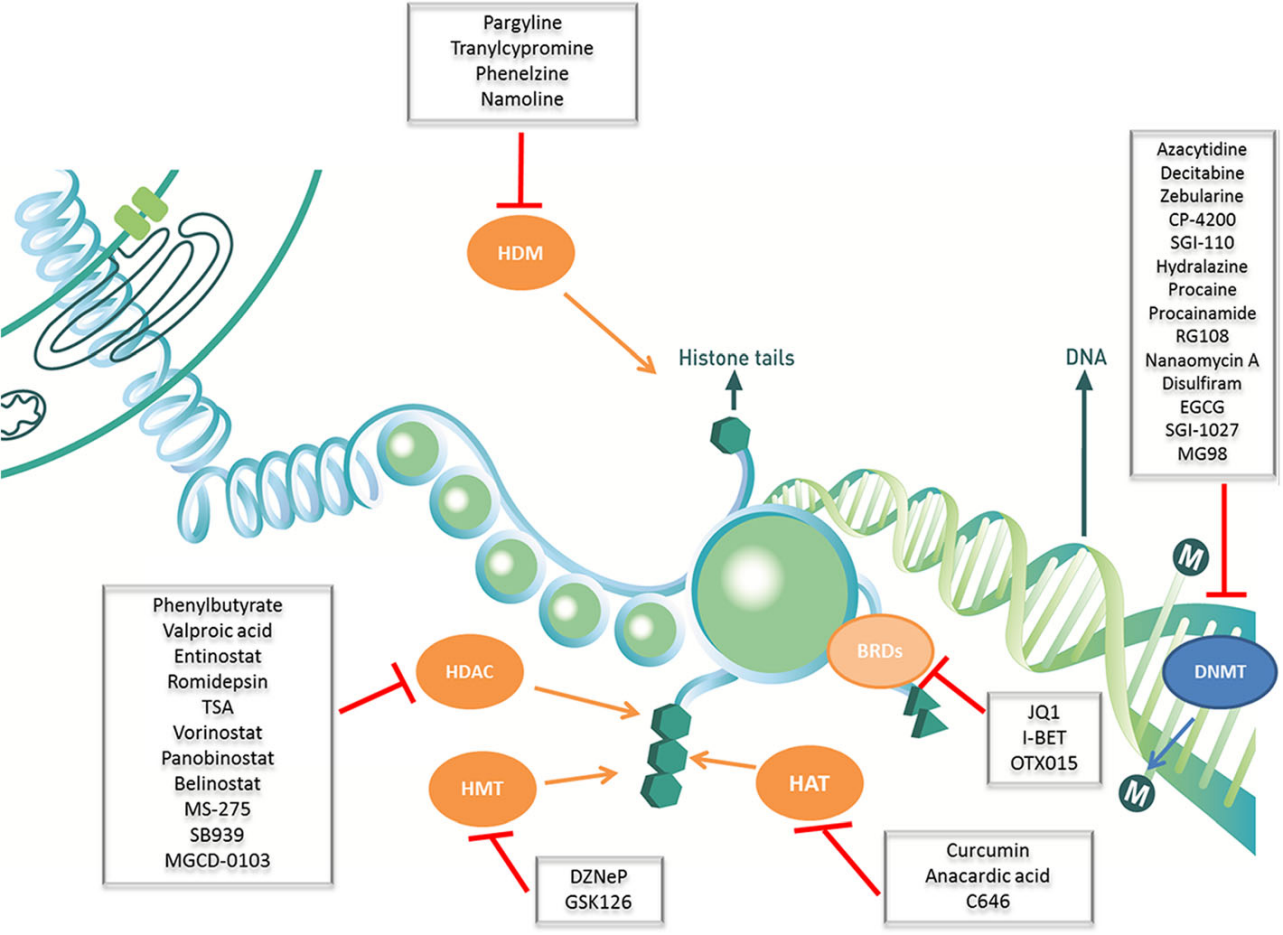
Epigenetic modifying drugs. This figure illustrates several epigenetic compounds classified accordingly to their respective epigenetic target that have been reported as having a role on PCa cell phenotype reversion either in pre-clinical or clinical assays

The interest in epigenetic modulators as targets for cancer therapy has been growing in recent years (Fig. 3) [61]. Indeed, six epigenetic compounds that target either DNA methylation or histone deacetylation have already been approved by the Food and Drug Administration (FDA) for cancer treatment (Table 1) [62–68]. Herein, we will focus on the advances of the use of DNMT inhibitors (DNMTi) and histone modulators for PCa therapy.

**Table 1:** Epigenetic drugs for cancer therapy approved by FDA

Drug	Comercial name	Company	Class	Year of approval	Treatment type	Cancer
5-Azacytidine	Vidaza®	Celgene Corporation	DNMTi	2004	Single agent	Myelodysplastic syndrome
5-Aza-2′-deoxycytidine	Dacogen®	Eisai	DNMTi	2006	Single agent	Myelodysplastic syndrome
Vorinostat/SAHA	Zolinza®	Merck	Pan-HDACi	2006	Single agent	Cutaneous T cell lymphoma
Romidepsin	Istodax®	Celgene Corporation	Class I HDACi	2009	Single agent	Cutaneous and peripheral T cell lymphoma
Belinostat	Beleodaq®	Spectrum Pharmaceuticals, Inc.	Pan-HDACi	2014	Single agent	Peripheral T cell lymphoma
Panobinostat	Farydak®	Novartis	Pan-HDACi	2015	Combination with bortezomid and examethasone	Multiple myeloma

### DNMT inhibitors

Among the epigenetic inhibitors, DNMTi are those in more clinically advanced stage of development. This family of compounds, depending on the mode of action, is divided in two classes: nucleoside and non-nucleoside inhibitors [69, 70].

Nucleoside analogues are composed of a modified cytosine ring that is attached to either a ribose or deoxyribose moiety and, therefore, can be incorporated into DNA or RNA, replacing cytosines. When incorporated into DNA during replication, these drugs covalently bind and capture DNMTs on the DNA strand. DNMTs are subsequently depleted due to passive demethylation during continuous replication. These agents induce cell death by obstructing DNA synthesis and/or inducing DNA damage through structural instability at the sites of incorporation [69, 71]. The two most studied nucleoside analogues are 5-azacytidine, a ribose nucleotide which is mostly incorporated into RNA interfering with protein synthesis, and 5-aza-2′-deoxycytidine which is incorporated preferentially into DNA. These DNMTi are approved for treatment of Myelodysplastic syndrome (MDS) and are currently in clinical trials in a range of other cancers [72]. However, azanucleosides have some pitfalls, including their higher instability and their short half-life owing to fast degradation by cytidine deaminase [69, 73]. Zebularine was shown to be more stable and less toxic than the 5-aza-nucleosides, since it was able to inhibit cytidine deaminase, it incorporates only in DNA via the ribonucleotide reductase pathway and induced minimal toxic effects in animals [74, 75]. This compound has proven anti-proliferative activity in cell lines and induces cancer cell death through alterations in DNA methylation status [74, 76–78]. CP-4200, an elaidic acid ester analog of 5-azacytidine, is a nucleoside transporter-independent drug which has shown superior efficacy to 5-azacytidine in an orthotopic acute lymphocytic leukemia (ALL) mouse tumor model [79] and was recently shown to overcome 5-azacytidine resistance mechanisms related to the cellular uptake in leukemia cells [80]. SGI-110 (guadecitabine) is a dinucleotide of 5-aza-2′-deoxycytidine and deoxyguanosine which confers relative resistance to cytidine deaminase and so an enhanced exposure to the active 5-aza-2′-deoxycytidine moiety. It was reported to be effective in inhibiting DNA methylation both in vitro and in vivo, and also acts as an immune modulator [81, 82]. Moreover, a phase I clinical trial showed good tolerance as well as clinical and biologic activity in MDS and acute myeloid leukemia (AML) patients [83].

One major limitation of nucleoside analogues is the requirement for DNA incorporation and active DNA synthesis, which limits the activity of these drugs in hypoproliferative cancers. This may be the major reason for their limited efficacy in the majority of solid tumors [84]. Because the nucleoside analogues are intrinsically cytotoxic, several efforts are being made to discover compounds that directly target DNMTs, without requiring prior incorporation into DNA. Presently, the non-nucleoside family includes compounds that have already been approved by FDA for non-neoplastic conditions, specifically hydralazine (anti-hypertensive drug), procaine (local anesthetic), and procainamide (anti-arrhythmic drug) and small molecules designed to directly block the active site of human DNMTs, like RG108. The major advantage of the former class is that the pharmacodynamic profile is well-known, and their adaptation for cancer therapy may be more cost-effective. The latter class, moreover, exhibits higher specificity, since the compounds are designed for direct enzyme inhibition [69, 73].

Hydralazine is a potent arterial vasodilator drug whose demethylating activity was suspected based in one of its secondary effects: the induction of Lupus-like syndrome [85–87]. Hydralazine was shown to induce tumor suppressor genes’ demethylation/reactivation in several cancer models and its activity is synergized with that of the histone deacetylase inhibitors (HDACi) valproic acid, both in vitro and in vivo [88–90]. Several clinical trials using hydralazine in combination with valproic acid in MDS and in solid tumors demonstrated no significant toxic effects [91–93]. Procaine and procainamide are two closely related small molecules that have been proposed to function as DNMTi also due to their ability to bind CpG-rich sequences, thereby interfering with DNMTs binding. Procainamide specifically inhibits DNMT1 but not DNMT3a and 3b, suggesting that this drug might be a highly specific inhibitor [94]. Both procaine and procainamide were reported to reduce DNA methylation in cancer cells [95, 96]. The antibiotic nanaomycin A was recently reported as a selective inhibitor of DNMT3b, with the ability to reduce methylation and induce expression of the tumor suppressor gene *RASSF1A* [97]. Disulfiram, a drug used in the clinics for the treatment of alcohol abuse, was described as a DNMT inhibitor with the ability to decrease the global levels of 5-methylcytosine, as well as to demethylate and reactivate the expression of epigenetically silenced tumor suppressor genes [98, 99]. SGI-1027, a quinoline-based compound, has demonstrated inhibitory activity against DNMT1, DNMT3a, and DNMT3b, possibly by interacting with the DNA substrate, which results in demethylation and reactivation of tumor suppressor genes [100]. RG108 was the first DNMTi designed to directly inhibit DNMT1 catalytic site. In fact, this compound was able to inhibit DNMT activity in a cell-free assay and to reduce global methylation levels in human cancer cells. [101–103]. Recently, this compound was also reported to induce differentiation of promyelocytic leukemia cells in combination with HDACi [104, 105]. MG98, a 20-bp anti-sense oligonucleotide, whose sequence is complementary to 3′-untranslated region (UTR) of DNMT1, was developed to block the translation of this enzyme [106]. Despite the DNMT1 inhibitory activity displayed in xenograft mouse models and in some patients, this compound did not achieve significant response in clinical trials [107–109]. Soy isoflavones (e.g., genistein) and their metabolites are also DNMTs inhibitors, with promising roles in cancer prevention and treatment [110]. The green tea polyphenol, (−)-epigallocatechin-3-*O*-gallate (EGCG) is an anti-tumoral agent that targets DNA methylation through DNMTs’ inhibition [111].

#### Pre-clinical activity of DNMT inhibitors in prostate cancer

In a pre-clinical assay, PCa cells chronically exposed to 5-aza-2′-deoxycytidine for 21 days, exhibited a marked decrease in tumor cell proliferation and AR reactivation, with concomitantly increased PSA protein levels. The restoration of AR-sensitized CRPC cells in xenograft models to the anti-androgen bicalutamide [112, 113]. 5-Aza-2′-deoxycytidine was able to decrease PCa stem-cellness and induce cell differentiation. In vitro and in vivo assays demonstrated that AR re-expression by 5-aza-2′-deoxycytidine led to in vitro and in vivo suppression of PCa stem cell proliferation, decreasing PCa tumorigenesis [114]. Zeburaline was able to restore GST-pi and GST-mu expression, both in vitro and in xenografts, enhancing the activity of brostallicin, a DNA minor groove binder with anti-cancer activity [115]. Additionally, PCa cell lines and xenografted mice exposed to procainamide demonstrated a reversion of *GSTP1* hypermethylation, with concomitant gene re-expression [96]. However, one study comparing the two non-nucleoside inhibitors with 5-aza-2′-deoxycytidine in PCa cell lines, demonstrated that 5-aza-2′-deoxycytidine was considerably more effective in demethylating and reactivating tumor suppressor genes [116]. Recently, synthesized procainamide conjugates proved to be more potent inhibitors of murine catalytic Dnmt3A/3L complex and human DNMT1, decreasing DU145 cell viability more efficiently than the parent compound [117]. Concerning RG108, we have demonstrated a dose- and time-dependent growth inhibition and apoptosis induction in LNCaP, 22Rv1, and DU145 PCa cell lines. This compound repressed DNMT activity and expression, reducing global DNA methylation in androgen-responsive PCa cells. Furthermore, exposure of LNCaP and 22Rv1 to RG108 significantly decreased promoter methylation levels of *GSTP1*, *APC*, and *RAR-β2*, although mRNA re-expression was only achieved for *GSTP1* and *APC* [118]. We have also recently demonstrated that hydralazine was able to restrain PCa cell growth and promote apoptosis in a time and dose dependent manner. Moreover, hydralazine decreased cellular invasiveness and induced cell cycle arrest and DNA damage in PCa cell lines. Additionally, PCa cells exposed to hydralazine exhibited lower *DNMT1*, *DNMT3a*, and *DNMT3b* mRNA levels as well as lower DNMT1 protein, which may have contributed to the observed decrease in *GSTP1*, B cell CLL/lymphoma 2 (*BCL2*) and *CCND2* promoter methylation levels, and concomitant gene re-expression. Importantly, hydralazine restored AR expression and upregulation of its target protein p21, in DU145 cells. The attenuation of tumor phenotype was particularly effective in the castration-resistant PCa cell line DU145, and this feature was associated with epidermal growth factor (EGF) receptor signaling disruption [119]. SGI-1027 was able to entirely deplete DNMT1 expression in LNCaP cells [100]. SGI-1027 and two analogues (paralmeta and metalmeta) inhibited effectively PC-3 proliferation and viability, at concentration of 100 μM [120]. Mahanine, a plant-derived carbazole alkaloid, inhibits DNMT activity inducing *RASSF1A* expression in LNCaP and PC-3 cells [121]. Moreover, this drug also inhibited LNCaP and PC-3 cells’ proliferation and induced apoptosis [122]. In a large study, the DNMT inhibitory effect of 1120 compounds was evaluated, from which 12 were selected for cytotoxicity tests in DU145 cells. Remarkably, the majority of the compounds with activity at low micromolar concentration displayed very limited cytotoxicity [123]. Genistein reduced *RARβ2*, *RASSF1A*, and *GSTP1* promoter methylation, entailing gene re-expression in PCa cell lines [124, 125]. Interestingly, this compound was able not only to reduce estrogen receptor-β (*ER-β*) promoter methylation, with corresponding increase in *ER-β* expression, but also decrease LNCaP and LAPC-4 cell proliferation [126]. Likewise, EGCG through DNMT1 activity inhibition induced re-expression of transcriptionally silenced genes in PCa cell lines [127, 128]. Finally, disulfiram exposure promoted PCa cells apoptosis and cell-cycle arrest, reduced tumor volume in xenograft mice, and restored expression of tumor suppressor genes, *APC*, *RAR-β* and *ER-β* through inhibition of DNMT activity [99, 129].

#### Clinical evaluation of DNMT inhibitors in prostate cancer

Although aberrant DNA promoter methylation is a major phenomenon in prostate carcinogenesis, there are only a few clinical trials testing DNMTi in PCa patients (Table 2). A phase II trial (NCT00384839) testing 5-azacytidine enrolled 36 PCa patients. PSA-doubling time (DT) less than 3 months was recorded in 19 patients, and the overall median PSA-DT was prolonged compared to baseline (2.8 vs. 1.5 months). One patient showed a 30 % PSA decline, whereas in 14 patients, only a slight PSA decline was observed. Grade 3 toxicities were reported and four patients had to stop therapy. DNA *LINE-1* methylation levels in plasma were also significantly decreased [130]. In a small phase II clinical trial, in which 14 patients with mCRPC were enrolled, 5-aza-2′-deoxycytidine was administered intravenously every 8 h at a dose of 75 mg/m^2^, every 5 to 8 weeks. Although well tolerated, only two patients showed disease stabilization with delayed time to progression for as long as 10 weeks [131].

**Table 2:** DNMT inhibitors in clinical trials for PCa

Drug	Clinical trial ID	Phase	Status	Protocol	Outcome	Ref.
5-Azacytidine (Vidaza)	NCT00384839	II	Completed	Patients with CRPC received 75 mg/m^2^ of 5-azacytidine for five consecutive days of a 28-day cycle. Patients were treated until clinical progression up to a maximum of 12 cycles. *n* = 36	5-Azacytidine modulates PSA (doubling time > 3 months) in 56 % of patients. Clinical progression-free survival of 12.4 weeks	[130]
5-Aza-2-deoxycytidine (decitabine)	–	II	Completed	14 patients with metastatic prostate cancer recurrent after total androgen blockade and flutamide withdrawal received three doses of 5-aza-2-deoxycytidine infusion (75 mg/m^2^). Cycles of therapy were repeated every 5 to 8 weeks. *n* = 14	Two of 12 patients evaluable for response had stable disease with a time to progression of more than 10 weeks. Modest clinical activity	[131]
5-Azacytidine, docetaxel, and prednisone	NCT00503984	I/II	Ongoing not recruiting	mCRPC patients, who progressed during or within 6 months of docetaxel chemotherapy, were eligible. In phase I, 5-azacytidine and docetaxel were alternately escalated in a three weekly cycle. All patients received prednisone 5 mg twice daily continuously. *n* = 22	Toxicity: myelosuppression Reduction in GADD-45 methylation on day 5	[273]
5-Azacytidine, phenylbutyrate	NCT00006019	II	Completed	Patients received 5-azacytidine subcutaneously on days 1–7 and phenylbutyrate I.v. over 1–2 h on days 8–12. Additional course was repeated every 21 to 28 days in the absence of disease progression or unacceptable toxicity. *n* = 20	Not available	

### Histone modulators (HDAC, HMTs, HDMi, and BET inhibitors)

Several compounds with the ability to modulate the expression of key enzymes involved in establishing (writers), removing (erasers), and maintaining (readers) epigenetic profiles have been identified as promising therapeutic tools for PCa (Fig. 3) [61, 132].

### HDAC inhibitors

HDACs overexpression is a common feature of human malignancies. Therefore, targeting HDACs has been a major research area in cancer therapy; although to date, the established clinical utility has remained rather modest. Thus far, various structurally different compounds have been tested in a broad range of cancers [133]. By altering the expression of several genes and/or function of several proteins, HDACi disrupt cancer cell pathways, such as cell proliferation, angiogenesis, differentiation, and apoptosis, culminating in cell cytotoxicity. In general, HDACi contain a zinc-binding domain connected by a straight chain linker to a capping group [134, 135]. HDCAi are chemically classified into different subgroups based on their structure: aliphatic acids (phenylbutyrate, sodium butyrate, and valproic acid), benzamides (mocetinostat and entinostat), cyclic peptides (romidepsin, largazole) and hydroxamic acids (trichostatin A (TSA), vorinostat/suberoylanilide hydroxamic acid (SAHA), belinostat, panobinostat) [136, 137]. Several, dietary phytochemicals (e.g., sulforaphane, phenethyl isothiocyanate) also inhibit HDAC activity suggesting anti-tumoral properties [138]. However, HDAC targeting is quite complex because they have multiple subclasses, some of which with yet unknown functions and mechanisms of action [133, 139]. Furthermore, enzymatic activity of HDACs is not restricted to histones, but extends to several other proteins [140].

### HAT inhibitors

Histone acetyltransferases inhibitors (HATi) have gained interest due to promising anti-cancer results in pre-clinical models of solid tumors [141]. Nevertheless, the discovery and design of selective HATi with high efficacy remains a challenge [142]. Currently, this family of compounds comprises four distinct classes: bisubstrate inhibitors, natural compounds and their analogues and derivatives, synthetic small molecules, and bromodomain inhibitors [142]. Curcumin, a component of *Curcuma longa* rhizome, is a specific inhibitor of p300/CREB-binding protein that inhibits acetylation of p53 in vivo [143]. This compound is currently under evaluation in clinical trials for colorectal (NCT01859858, NCT00745134, NCT02724202, and NCT02439385) and breast (NCT01740323 and NCT01975363) cancers. CTK7A (hydrazinobenzoylcurcumin) is a water-soluble inhibitor of p300 and several other proteins that reduce xenograft tumor growth in mice [144]. Anacardic acid, a non-specific HATi of p300, isolated from the liquid of cashew nut shells, also demonstrated anti-cancer activity through modulation of nuclear factor kappa B (NF-kB) pathway [145]. Garcinol, a micromolar inhibitor of p300 and P300/CBP-associated factor (PCAF) obtained from *Garcinia indica*, displays anti-tumor activity by inducing apoptosis and inhibiting autophagy of human cancer cells [146, 147]. Plumbagin, a potent KAT3B/p300 inhibitor isolated from *Plumbago rosea*, decreased tumor cell growth, angiogenesis, and invasion in several cancer models [148–151]. With a similar scaffold, Embelin, isolated from *Embelia ribes*, specifically inhibits H3K9 acetylation and also displays anti-tumor activity [152–154]. NK13650A and NK13650B are two novel compounds with anti-cancer activity that have been extracted from a *Penicillium* strain, demonstrating a strict p300 selectivity [155]. C646 is synthetic small selective molecule inhibitor of p300/CBP that was shown to induce apoptosis in cancer cells through inhibition of AR and NF-kB pathway [156, 157]. Two other synthetic compounds, NU9056 and TH1834, are specific micromolar inhibitors of TIP60 (KAT5) acetyltransferase activity [158, 159].

### HMT and HDM inhibitors

HMTs and HDMs are emerging as a novel field of epigenetic actionable molecules with clinical interest. Several new compounds are currently under evaluation to assess their specificity for targeted epigenetic therapy and its anti-cancer effectiveness [160–162]. These compounds are thought to be more attractive than HDACi because they can eliminate selective histone marks, which in turn might enable a better tailored therapy, minimizing undesirable side effects.

Among histone methyltransferase inhibitor (HMTi), 3-dezaneplanocin-A (DZNeP) stands as a *S*-adenosyl-l-homocysteine (AdoHcy) hydrolase inhibitor which converts adenosyl-l-homocysteine, produced by methyltransferases, in adenosine and homocysteine. *S*-Adenosyl-methionine (AdoMet), a methyl donor for methylation reactions, is metabolized to AdoHcy by methyltransferases. By increasing AdoHcy levels, DZNeP inhibits methyltransferases. This compound was first reported as EZH2 inhibitor, decreasing H3K27 trimethylation, but is currently considered a global HMTi [163, 164]. DZNeP downregulates EZH2, reactivates several tumor suppressor genes inhibited by polycomb repressive complex 2 (PRC2), and inhibits cancer cell phenotype [163, 165, 166]. GSK126 is a small molecule that inhibits methyltransferase activity of both wild-type and mutant EZH2, is independent of substrate, and, more importantly, is extremely selective against other methyltransferases and/or other proteins [167, 168]. Like DZNeP, this compound reduces global H3K27me3 levels and induces expression of silenced PRC2 target genes. GSK126 reduced the proliferation of cancer cells lines and inhibited tumor growth in xenografts [168–170]. EPZ-6438 (tazemetostat) is also an effective and orally bioavailable EZH2 inhibitor with anti-cancer activity [171]. Other novel EZH2 inhibitors are currently under clinical trial, namely CPI-1205 (NCT02395601), E7438 (NCT01897571), tazemetostat (NCT02601937 and NCT02601950), and GSK2816126 (NCT02082977).

LSD1 inhibitors represent the family of histone demethylase inhibitors (HDMi) most studied thus far, and the majority of the assays were performed with non-selective amine oxidase (MAO) inhibitors (pargyline, tranylcypromine, and phenelzine). These compounds irreversibly react with flavin adenine dinucleotide (FAD) through a radical mechanism, forming a tetracyclic adduct, and were originally designed for treatment of psychiatric illnesses. Presently, they are under investigation for cancer therapy due to their ability to block LSD1 [55, 172–174]. Namoline was reported as a selective and reversible inhibitor of LSD1, with in vitro and in vivo activity, that might interfere with global histone methylation levels [175].

### BET inhibitors

Bromodomain (BET) proteins bind to acetylated histones, increase proliferation, and may lead to overexpression of several oncogenes such as MYC [176]. JQ1 and I-BET (I-BET762 or GSK525762) are novel compounds that inhibit bromodomain proteins competing with its binding to histone acetylated lysine residues, which results in the displacement of BET proteins from acetylated chromatin [177]. Both compounds were shown to induce cellular differentiation, senescence, and apoptosis [178]. JQ1 showed selectivity for the BET family, with higher affinity for Bromodomain-Containing Protein 4 (BRD4) and demonstrated anti-tumor activity in several cancer cell types [179–181]. I-BET, also a diazepine-based compound with proved in vitro and in vivo anti-cancer activity, is currently in phase I clinical trials for hematological malignancies (NCT01943851) and solid tumors (NCT01587703) [182, 183]. OTX015, a novel oral inhibitor of BRD2/3/4, derivative of JQ1 that was originally developed for the treatment of inflammatory bowel disease, also demonstrated in vitro and in vivo anti-neoplastic efficacy and is currently in phase I clinical trials for hematological malignancies (NCT01713582) and several solid tumors (NCT02259114), as well as in a phase IIa trial for glioblastoma multiform (NCT02296476) [177, 184, 185]. I-CBP112, that targets CBP/p300 bromodomains, induces differentiation, cell cycle arrest, and suppresses tumor proliferation [186, 187].

#### Pre-clinical activity of HDACi in prostate cancer

Several HDACi demonstrated encouraging results in pre-clinical phase studies, showing promise as candidates for future clinical trials.

Concerning the aliphatic acids family, exposure to sodium butyrate induced growth inhibition and increased differentiation and apoptosis of PC-3 and DU145 cells [188, 189]. Remarkably, treatment with sodium butyrate also induced H2B acetylation, and methylation on multiples lysine residues, as well as phosphorylation of Thr19, in DU145 cells [190]. Recently, this compound was shown to stimulate the morphological and molecular differentiation of LNCaP cells via inhibition of T-type Ca^2+^ channels [191]. Valproic acid (VPA) also reduced cell viability and induced apoptosis in vitro and was able to reduce tumor growth in xenograft models [192]. Moreover, this compound inhibited epithelial-mesenchymal transition (EMT) and invasion abilities of PC-3 cells by decreasing SMAD4 protein expression and upregulating the metastasis suppressor gene N-myc downstream regulated gene-1 (*NDRG1*), respectively [193, 194]. In a TRAMP model of PCa treated with VPA, decreased tumor growth and invasiveness correlated with the re-expression of *CCND2*, a frequently silenced gene in PCa [195]. Remarkably, this compound also induced AR and E-cadherin expression in PCa cell lines [196].

Among hydroxamic acids, vorinostat/SAHA demonstrated the ability to decrease PCa cell lines proliferation and to reduce tumor growth in vivo [197, 198]. Panobinostat also induced cell cycle arrest and DNA damage and reduced PCa tumor growth in vivo [199]. Moreover, the exposure of PCa cells to this compound lead to a decrease in *AR* levels and reversed resistance to hormone therapy in castration-resistant PCa cell lines [200]. Belinostat showed pronounced anti-tumor effects in androgen-responsive PCa cell lines increasing p21, p27, and p53 protein expression and leading to G2/M cell cycle arrest [201]. It also reduced the migration of PCa cells, increasing the expression of tissue inhibitor of metalloproteinase-1 (TIMP-1). Moreover, it decreased the expression of oncogenic proteins, such as mutant P53 and ERG. Notably, the cytotoxic activity of this compound was preferentially directed against tumor cells [202].

Concerning the cyclic peptides family, mice inoculated with the 22Rv1 cell line exposed to romidepsin not only experienced reduced metastasis formation but also induces a 61 % survival increase [203]. Largazole and 2-epi-largazole are potent class I-selective HDACi, purified from marine cyanobacteria, that decrease LNCaP and PC-3 cell viability [204].

The benzamide derivative MS-275 increased H3 acetylation, p21 protein expression, and induced growth arrest in LNCaP and PC-3 cells and apoptosis in DU145 cells. Moreover, MS-275 reduced tumor growth in xenograft mice [205], particularly when acting synergistically with radiation therapy [206]. This drug also lead to H3K4 methylation upregulation, inducing re-expression of tumor suppressor and cell differentiation genes [207].

Sulforaphane, an isothiocyanate isolated form broccoli, suppressed PCa tumor cell growth in male nude mice and significantly correlated with decreased HDAC activity in prostate tissue and mononuclear blood cells. Moreover, in human subjects, the consumption of BroccoSprouts (68 g) also inhibited HDAC activity in peripheral blood mononuclear cells [208]. Importantly, another study demonstrated that sulforaphane effects are selective, since it more potently induced cell cycle arrest apoptosis and acetylation of H3 at *P21* promoter and inhibited HDAC activity in benign hyperplasia (BPH1) and cancer (LNCaP and PC-3) PCa cells than in the normal cell line PrEC [209]. It was also reported that this compound destabilizes AR by hyperacetylating HSP90, via restraining HDAC6, leading to AR proteasomal degradation [210]. Recently, it was shown that sulforaphane was able to decrease MYC expression, the activity of aldehyde dehydrogenase 1 (ALDH1), CD49f + fraction enrichment and the efficiency of sphere forming, all characteristics of PCa stem cells [211]. Phenethyl isothiocyanate (PEITC), another isothiocyanate, suppressed PCa progression in transgenic adenocarcinoma of mouse prostate mice by induction of autophagic cell death and overexpression of E-cadherin [212]. Another study demonstrated that PEITC suppressed androgen-responsive tumor growth in vivo, possibly by downregulation of integrin family proteins (β1, α2, and α6) and tumor platelet/endothelial cell adhesion molecule (PECAM-1/CD31) [213]. This compound also promoted apoptosis and cell cycle arrest and inhibited invasion and on in vitro and in vivo models of PCa [214–216]. Like sulforaphane, PEITC repressed AR transcription and expression [217].

New specific HDAC1 inhibitors designed and synthetized using click chemistry revealed anti-proliferative activity in DU145 cells at micromolar concentrations [218]. A specific inhibitor of HDAC6, *N*-hydroxy-4-(2-[(2-hydroxyethyl)(phenyl)amino]-2-oxoethyl)benzamide (HPOB) decreased viability of LNCaP cells without affecting cell death or causing DNA damage. Furthermore, this compound inhibited HDAC6 deacetylase activity but not its ubiquitin-binding activity and incremented the cell death effect of SAHA, etoposide, and doxorubicin [219]. A novel compound, 3-hydroxypyridin-2-thione (a non-hydroxamate chemotype), was able to reduce expression of HDAC6 and 8 and suppress viability of LNCaP cells. This might be due, in part, to induced hyperacetylation of Hsp90 that subsequently attenuates interactions of key proteins essential for LNCaP cells survival, such as AR [220]. New class II-selective hydroxamate inhibitors, that target HDAC4 and HDAC6, were effective in decreasing cell proliferation and inducing cell cycle arrest at G1 phase and nuclear histone acetylation of PC-3 and LNCaP cells [221]. Benzothiazole-containing analogues of vorinostat/SAHA compounds displayed not only anti-proliferative effects in PC-3 cells but it also reduced tumor growth in a PC-3 mouse xenograft with efficacy equivalent to vorinostat/SAHA [222].

Development of hybrid compounds that could modulate multiple targets with superior efficacy and fewer side effects than current single-target drugs is underway [133]. A new set of HDACi were generated to selectively accumulate in PCa cells. A non-steroidal anti-androgen scaffold based on cyanonilutamide was incorporated into a prototypical HDACi (vorinostat/SAHA) pharmacophore, creating an AR-HDACi which will first engage AR, selectively accumulate, and then released to engage HDACs. These compounds demonstrated improved inhibition of all HDACs’ activity compared to vorinostat/SAHA alone and were able to simultaneously antagonize AR. Moreover, they displayed anti-proliferative activity in AR-expressing cell lines [223]. Another hybrid compound that resulted from the combination of methotrexate and hydroxamate (methotrexate-caproic hydroxamic acid) reduced HDAC activity and decreased viability of PC-3 cells [224]. Additionally, a new drug, VPA–GFLG-iRGD, which conjugates VPA with a cell penetrating peptide (iRGD) and a lysosomally degradable tetrapeptide (–GlyPheLeuGly–, –GFLG–), induced a significant decrease in the proportion of DU145 cells in G2 phase with increased cytotoxicity. This might be related with RGB induced blockage of α_ν_β_3_ and α_ν_β_5_ integrin on DU145 cell surface [225]. Likewise, the synthesis of dual-acting histone deacetylase (vorinostat/SAHA) and topoisomerase II inhibitors (anthracycline daunorubicin) resulted in decreased proliferation of DU145 cells [226]. Recently, WJ35435, a hybrid vorinostat/SAHA and DACA (topoisomerase inhibitor) molecule with anti-HDAC activity, showed a more potent anti-cancer effect, inducing more potent cell cycle arrest, DNA damage and apoptosis, than either agent alone, in PC-3 and DU-145 cells. Furthermore, this compound revealed anti-tumor activity in vivo and, importantly, it did not affect benign prostate cells [227]. Recently, CUDC-101, which resulted from the incorporation of HDAC inhibitory functionality into the pharmacophore of epidermal growth factor receptor (EGFR) and human epidermal growth factor receptor 2 (HER2)/NEU inhibitors [228], was able to reduce *AR* and *AR-v7* expression, PCa cell proliferation in vitro and in vivo [229]. This compound is currently in phase I trial in solid tumors (NCT01702285).

#### Clinical trials testing HDACI in prostate cancer

Several HDACi are under clinical trial for PCa treatment (Table 3). A phase II clinical trial (NCT00330161) with vorinostat/SAHA was conducted in mCRPC patients with disease progression and previously treated with chemotherapy [230]. Patients were daily treated with orally administrated 400 mg vorinostat/SAHA. The best objective response was stable disease in 2 out of the 27 (7 %) patients enrolled in this trial. Median time to progression was 2.8 months, with a median overall survival of 11.7 months. Grade 3 or 4 toxicities (fatigue, nausea, vomiting, anorexia, diarrhea, and weight loss) were experienced by 48 % of patients and 11 (41 %) actually discontinued therapy due to toxicity. Thus, vorinostat/SAHA at this schedule had marginal therapeutic efficacy, and this might be associated with the substantial toxicities described. Recently, a phase II clinical trial evaluated the efficacy of panobinostat in CRPC patients (NCT00667862) with disease progression after chemotherapy [231]. The rate of progression-free survival (PFS) at 24 weeks was set as primary endpoint. Thirty-five patients received 20 mg/m^2^ of panobinostat intravenously on days 1 and 8 of a 21-day cycle. No objective responses were documented. Four patients (11.4 %) did not show progression of disease at 24 weeks. All patients experienced grade 3 and 4 toxicities. Therefore, it was concluded that PCa treatment with panobinostat alone was insufficient to achieve clinical efficacy [231]. A phase II study with romidepsin was conducted in 35 metastatic CRPC patients (NCT00106418). Romidepsin was administrated intravenously at 13 mg/m^2^ on days 1, 8, and 15 of a 28-day cycle [232]. Partial response confirmed by radiology and PSA decline was achieved in two patients. Eleven patients, however, experienced significant drug toxicity and discontinued therapy. With this drug schedule, romidepsin demonstrated minimal anti-tumor activity in mCRPC patients.

**Table 3:** Histone modifying drugs in clinical trials for PCa

Drug	Clinical trial ID	Phase	Status	Protocol	Outcome	Ref.
Vorinostat/SAHA	NCT00330161	II	Completed	Metastatic PCa with disease progression on prior chemotherapy received 400 mg vorinostat/SAHA orally each day. Disease progression measured at 6 months. *n* = 27	Toxicity: significant toxicities including fatigue, nausea. IL-6 (Interleukin 6) was higher in patients with toxicity. 7 % patients achieved a stable disease state. No PSA decline >50 % observed. Median time to progression and overall survival were 2.8 and 11.7 months, respectively. Significant toxicities reported.	[230]
Vorinostat/SAHA	NCT00005634	I	Completed	Patients with advanced or metastatic solid tumors that have not responded to previous therapy received vorinostat/SAHA I.v. on days 1–3 every 21 days. *n* = 45	Determine the tolerability, pharmacokinetic profile, and biologic effects of the drug. Not available	
Vorinostat/SAHA and docetaxel	NCT00565227	I	Terminated due to toxicity	Patients with advanced and relapsed tumors received oral vorinostat/SAHA for the first 14 days of a 21-day cycle, with docetaxel I.v. on day 4 of each cycle. *n* = 12	Toxicity: neutropenia, peripheral neuropathy, and gastrointestinal bleeding. The combination of vorinostat/SAHA and docetaxel was poorly tolerated. No responses were identified.	[274]
Vorinostat/SAHA and doxorubicin	NCT00331955	I	Completed	Patients receive oral vorinostat/SAHA twice daily for 5 doses on days 1–3, 8–10 and 15–17 and doxorubicin I.v. on days 3, 10, and 17 very 28 days for up to 6 courses. *n* = 32	Partial response was achieved in one of the two PCa patients enrolled.	[275]
Vorinostat/SAHA and androgen deprivation therapy (ADT)	NCT00589472	II	Completed	Localized PCa patients received neo-adjuvant vorinostat/SAHA with oral bicalutamide with either I.M. leuprolide or subcutaneous goserelin acetate administered for up to 8 weeks or until the day of surgery. *n* = 19	Determine the rate of pathologic complete response in patients with localized PCa treated with ADT and vorinostat/SAHA before radical prostatectomy measuring androgens in blood. Not available	
Vorinostat/SAHA and mTOR inhibitor temsirolimus	NCT01174199	I	Ongoing, not recruiting	Metastatic PCa patients received oral vorinostat once daily on days 1–14 and temsirolimus intravenously on days 1, 8, and 15 of a 21-day cycle. *n* = 13	Determine the safety, tolerability, partial and complete objective response rates, progression-free survival and overall survival, and PSA response. Not available	
Panobinostat	NCT00667862	II	Completed	I.v. panobinostat (20 mg/m2) was administered to CRPC patients on days 1 and 8 of a 21-day cycle. Disease progression measured at 24 weeks. *n* = 35	Toxicity: fatigue, thrombocytopenia, nausea 14 % patients demonstrated a decrease in PSA but none >50 %. No clinical activity.	[231]
Panobinostat (LBH589), docetaxel, and prednisone	NCT00663832	I	Completed	CRPC patients received oral panobinostat (20 mg/m2) on days 15 for 2 consecutive weeks. On the other arm, patients received oral panobinostat (15 mg/m2) with docetaxel I.v. (75 mg/m2) every 21 days and oral prednisone (5 mg) twice every day of a 21-day cycle. *n* = 16	Toxicity: dyspnea and neutropenia Panobinostat in combination with docetaxel and prednisone in patients with CRPC resulted in 63 % of patients with >50 % decline in PSA levels. No relevant anti-tumor activity.	[276]
Panobinostatbicalutamide	NCT00878436	I/II	Completed	Men with CRPC received treatment with bicalutamide (50 mg PO) daily with oral panobinostat at 2 dose levels (20 or 40 mg). Minimum treatment was 3 weeks. *n* = 9	Toxicity: thrombocytopenia In 2 patients, it was registered a >50 % PSA decline by 9 months of therapy; and 3 patients presented stable PSA levels.	[277]
Panobinostat docetaxel and prednisone	NCT00493766	I	Terminated because of a strategic decision	In one arm, oral panobinostat alone is given to patients with progressing hormone refractory prostate cancer. In the other arm, oral panobinostat along with I.v. docetaxel and oral prednisone is administered. *n* = 16	Toxicity: dyspnea, neutropenia, fatigue. Exposure to oral panobinostat was similar with and without docetaxel.	
Panobinostat, docetaxel, and prednisone	NCT00419536	I	Terminated because of a strategic decision	Not available	Determine maximum tolerated dose of panobinostat and to characterize the safety, biological activity, and pharmacokinetic profile.	
Panobinostat, radiotherapy	NCT00670553	I	Completed	Not available. *n* = 7	Establish toxicity, tolerability, and safety of oral panobinostat when given in combination with radiotherapy. Not available	
Romidepsin	NCT00106418	II	Completed	mCRPC patients received romidepsin (13 mg/m2) intravenously on days 1, 8, and 15 every 21-day cycle. Disease progression measures at 6 months. *n* = 35	Toxicity: nausea, fatigue 2 patients reached a confirmed radiological partial response of over 6 months, in addition to >50 % PSA decline. 11 patients had to discontinue the therapy due to toxicity. Romidepsin demonstrated minimal anti-tumor activity in chemonaive patients with CRPC.	[232]
Romidepsin	NCT00106301	II	Completed	Patients with CRPC were continued at the same dose of romidepsin as in the previous study, which could have been 13 mg/m2 or a reduced dose of 10 mg/m2, on days 1, 8, and 15 of each 28-day cycle. *n* = 2	Evaluate adverse effects and effect of romidepsin and evaluate the time of disease progression. Not available	
Romidepsin in solid tumors with liver dysfunction	NCT01638533	I	Currently recruiting patients	Patients with recurrent prostate carcinoma receive romidepsin I.v. on days 1, 8, and 15. Courses repeat every 28 days in the absence of disease progression or unacceptable toxicity. *n* = 132	Establish the safety and tolerability, pharmacokinetics, and maximum tolerated dose. Not available	
Pracinostat	NCT01075308	II	Completed	Recurrent or mCRPC patients received pracinostat orally (60 mg) 3 times a week for 3 consecutive weeks followed by 1 week off-dosing of a 28-day cycle. *n* = 32	Toxicity: fatigue, neutropenia 2 patients achieved a decline >50 % of PSA. In patients with measurable disease, there were no objective responses, while 7 patients had stable disease lasting 1.7 to 8 months.	[233]
Valproic acid	NCT00670046	II	Not provided	Non-metastatic with biochemical progression PCa patients received oral valproic acid twice daily for up to 1 year in the absence of disease progression or unacceptable toxicity. *n* = 50	Percentage of patients exhibiting observed or predicted PSA doubling time >10 months after initiation of the study. Not available	
Valproic acid and bevacizumab	NCT00530907	I	Completed	Bevacizumab was administered at escalating dosages of 2.5–11 mg/kg on days 1 and 15, and oral valproic acid at dosages of 5.3–10 mg/kg on days 1–28, every 28. *n* = 57	Toxicities: grade 3 altered mental status (*n* = 2), related to valproic acid. Bevacizumab 11 mg/kg given on days 1 and 15 and valproic acid 5.3 mg/kg daily were the recommended phase II dosages. Stable disease ≥6 months were reported in 4/57 of patients. Of the 39 patients evaluated for histone acetylation, 2 of 3 (67 %) patients with stable disease ≥6 months showed histone acetylation, while 8 of 36 (22 %) without stable disease ≥6 months demonstrated histone acetylation (*p* = 0.16). Patients with hypertension had improved overall survival.	[278]
Sulforaphane	NCT01228084	II	Completed	Patients with biochemical (PSA) recurrent PCa received 200 μmoles/day sulforaphane-rich extracts during 20 weeks. *n* = 20	1 patient experienced a ≥50 % PSA decline and 7 patients had PSA declines >50 %. No grade 3 events reported.	[234]
Sulforaphane	–	II	Completed	PCa patients with increasing PSA levels after prostatectomy orally received 60 mg of sulforaphane or placebo for 6 months. *n* = 78	Sulforaphane-treated patients presented 86 % longer PSA-DT than the placebo group. Increases >20 % of PSA levels higher in the placebo group (71.8 %) compared to the sulforaphane-treated group (44.4 %)	[235]
MGCD-0103 and docetaxel	NCT00511576	I	Terminated	Patients received escalating doses of oral MGCD-0103 in combination with two fixed doses of I.v. docetaxel (60 mg/m2 and 75 mg/m2). *n* = 54	Determine the maximum tolerated dose, dose limiting toxicitie,s and safety profile of escalating doses of oral MGCD-0103 in combination with two fixed doses of docetaxel. Not available	
Curcumin	NCT02064673	II	Recruiting	PCa patients with localized disease who were submitted to a radical prostatectomy received oral curcumin or placebo 500 mg twice a day for 6 months. *n* = 600	Determine recurrence-free survival as total PSA <0.2 ng/ml. Not available	
Curcumin, prednisone, and docetaxel	–	II		Patients with progressing CRPC and a rising PSA received docetaxel/prednisone for 6 cycles in combination with curcumin, 6000 mg/day (day −4 to day +2 of docetaxel). *n* = 30	Decreased PSA levels were observed in 59 % of patients and 40 % of evaluable patients presented a partial response. The regimen was well tolerated.	[279]
Curcumin and radiotherapy	NCT01917890	Not provided (pilot)	Completed	PCa patients undergo 74 Gy radiotherapy 5 times a week for 7–8 weeks and take 3 g of curcumin vs placebo. *n* = 40	The change in urinary symptoms across the 20-week period differed significantly between groups (*p* = 0.011) and patients in the curcumin group experienced much milder urinary symptoms compared with the placebo group. Curcumin could not reduce the severity of bowel symptoms or other treatment-related symptoms. PSA levels were reduced to below 0.2 ng/ml in both groups.	[280]
Curcumin and taxotere	NCT02095717	II	Recruiting	mCRCP patients receive taxotere plus curcumin capsule vs taxotere plus placebo. *n* = 100	Assess time to progression of metastatic disease by tumor response rate, increase in PSA levels (≥25 % and ≥2 ng/ml increase) or the appearance of new lesions metastatic. Not available	
Phenelzine	NCT02217709	II	Recruiting	Recurrent non-metastatic PCa received phenelzine daily and orally during 12 months. *n* = 46	Determine biochemical recurrent prostate cancer by PSA decline to ≥50 % following at least 12 weeks of treatment. Not available	
Phenelzine and docetaxel	NCT01253642	II	Recruiting	PCa patients with progressive disease after first-line therapy with docetaxel received phenelzine orally once a day on days −7 to −4 and twice a day on days −3 to 21 and docetaxel I.v. on day 1. Treatment repeats every 21 days for at least 12 weeks. *n*n = 30	Determine the proportion of patients who experience a PSA decline of at least 30 % and duration of progression-free survival. Not available	
OTX015	NCT02259114	IB	Recruiting	Advanced solid tumors including CRPC. Patients divided in two regimens: (1) continuous, once daily for 21 consecutive days and (2) once daily on days 1 to 7, repeated every 3 weeks (1 week on/2 weeks off). *n* = 98	Determine maximum tolerated dose and the number of dose limiting toxicity. Not available	

A recent phase II trial with pracinostat (NCT01075308), an orally active hydroxamic acid, enrolled 32 CRPC patients, which received 60 mg three times per week, on alternate days, for three weeks, followed by one-week resting period. The drug was well tolerated, and confirmed PSA response was noted in 6 % of the patients whereas stable disease (from 1–8 months) was achieved in six patients. During treatment, 64 % of patients demonstrated a conversion from unfavorable to favorable circulating tumor cells (CTC) profile [233]. A phase II trial (NCT01228084) evaluated the anti-tumor efficacy, safety, pharmacokinetics, and pharmacodynamics of sulforaphane-rich extracts (200 μmoles/day during 20 weeks) in 20 patients with biochemically (PSA) recurrent PCa. PSA decline was used as primary endpoint. One patient experienced ≥50 % PSA decline, and seven patients had PSA declines less than 50 %. No grade 3 events were reported [234]. A double-blinded, randomized, placebo-controlled multicenter trial of sulforaphane-enrolled 78 PCa patients with increasing PSA levels after radical prostatectomy. Sulforaphane was orally administered daily (60 mg) for six months followed by two months without treatment. Patients treated with sulforaphane presented 86 % longer PSA-DT than the placebo group. Furthermore, changes in PSA levels (increases >20 %) were significantly higher in the placebo group (71.8 %) compared to the sulforaphane-treated group (44.4 %) [235].

Considering these results, HDACi alone did not demonstrate promising results for PCa therapy. Their fast excretion and off-target toxicity allied to their inability to significantly accumulate in solid tumors might be responsible for its lack of efficacy against PCa. Therefore, investigation of new HDACi should be focused on improving tumor cell selectivity and tissue distribution.

#### Pre-clinical activity of HATi in prostate cancer

Exposure of PCa cells to curcumin decreased cell proliferation, increased apoptosis and downregulated several important metastasis-promoting genes, including cyclooxygenase-2 (*COX2*), Secreted Protein Acidic And Cysteine Rich (*SPARC*) and EGF-containing fibulin-like extracellular matrix protein (*EFEMP*) [236]*.* This compound also abrogated HGF-mediated increase of vimentin in DU145 cells by downregulating the expression of phosphorylated c-Met, extracellular signal-regulated kinase and Snail, therefore inhibiting EMT [236]. Additionally, it reduced metastasis formation *in vivo* [237]. Curcumin was also able to demethylate and restore neurogenin 1 (*Neurog1*) expression and decrease methyl CpG binding protein 2 (*MeCP2*) binding to *Neurog1* promoter in LNCaP cells [238]. CTK7A targets AR amino-terminal domain leading to its inhibition and to decreased proliferation of androgen-sensitive and castration-resistant AR-positive PCa cells. Moreover, it suppressed tumor growth in a xenograft model of CRPC [239]. Anacardic acid decreased cell proliferation and induced G1/S cell cycle arrest and apoptosis of LNCaP cells. The anti-growth effects of this compound in PCa could be mediated by induction of p53 and p21 protein expression and downregulation of *AR* [240]. Garcinol inhibited autophagy and colony formation ability, induced apoptosis of human PCa cells, and reduced tumor volume in a xenograft mouse model [241, 242]. Importantly, apoptosis seemed to be mediated by garcinol-mediated downregulation of NF-kB signaling [242]. Likewise, in PCa cell lines, plumbagin decreased cell proliferation and increased mitochondria-mediated apoptosis and autophagy through inhibition of PI3K/Akt/mTOR pathway and SIRT1, respectively [243]. These effects were particularly manifest in BRCA1/2-negative CRPC cells. This compound also seems to target PCa stem cells [244]. Furthermore, Embelin was shown to inhibit cell growth, migration, and invasion of PCa cell lines through modulation of Akt signaling and GSK-3β activation [245, 246]. This compound potentiated radiotherapy for tumor growth suppression (in vitro and in vivo) and increased the anti-proliferative and the apoptotic effects of anti-androgen therapy leading to AR downregulation [247, 248]. Accordingly, NK13650A inhibited AR mediated transcriptional activation in both hormone-naïve and castration-resistant PCa cells [155]. On the other hand, C646 induced caspase-dependent apoptosis and decreased the migration and invasion capacity of PCa cells [157]. Interestingly, TIP60 which may function as AR co-activator is overexpressed in PCa tissues and significantly correlates with disease progression [249]. NU9056 inhibits TIP60 activity, as well as *AR* and *PSA* expression, reducing cell viability and inducing apoptosis via caspases 3 and 9 activation in PCa cell lines. Remarkably, CRPC cell lines were more sensitive to NU9056 than hormone-naïve cells [158]. Both NU9056 and TH1834 sensitized PCa cells to radiation therapy [158, 159].

Two clinical trials with curcumin are now recruiting PCa patients (NCT02064673 and NCT02095717).

#### Pre-clinical activity of HMTi and HDMi in prostate cancer

Exposure of PCa cells to DZNeP resulted in cell cycle arrest in LNCaP and increased apoptosis in DU145 cells and diminished its invasion capacity. Moreover, this compound reduced tumor growth in mice and decreased PCa stem cells self-renewal [250]. GSK126 inhibited either polycomb-dependent or independent activity of EZH2 in PCa cells [251]. EPZ005687 demonstrated dose-dependent inhibition of H3K27me3 in PCa cells [252]. A-366 is a potent G9A and GLP inhibitor which efficiently reduces H3K9me2 in PC-3 cells, at micromolar concentrations [253]. CARM1 (PRMT4) inhibitors [1-benzyl-3,5-bis-(3-bromo-4-hydroxybenzylidene)piperidin-4-one and its analogues] significantly reduced *PSA* promoter activity in LNCaP cells in a dose-dependent fashion [254]. Currently, there are no clinical studies involving HMTi in PCa.

Pargyline decreased demethylation of H3K9 by LSD1, which co-localizes with AR, therefore inhibiting androgen target genes re-expression in PCa [55]. Furthermore, this LSD1 inhibitor reduced migration and invasion ability and inhibited EMT transition in vitro and in vivo*.* Suppression of EMT transition was apparent through increased E-cadherin expression, and N-cadherin, and vimentin downregulation. This drug was also able to reduce PSA expression both in vitro and in vivo, delaying CRPC onset [255]. Pargyline and tranylcypromine induced cell cycle arrest at G1 and increased apoptosis of LNCaP cells [256]. LNCaP cells and xenograft models treated with namoline, displayed reduced cell viability, and tumor volume. This compound was proposed as a potential therapeutic agent against hormone-sensitive PCa, since it induced silencing of AR-regulated genes [175]. Because LSD1 and JMJD2 are coexpressed and colocalized with AR in PCa cells, there have been efforts to synthetize pan-demethylase inhibitors that might simultaneously inhibit LSD1 and JmjC KDMs. Several of these compounds induced apoptosis, arrested cell cycle at G1, and decreased proliferation and migration of LNCaP cells [257].

Finally, two clinical trials will be conducted with the non-specific MAO inhibitor phenelzine, alone (NCT02217709) or in combination with docetaxel (NCT01253642).

#### Pre-clinical activity of BET inhibitors in prostate cancer

I-BET762 decreased PCa cell lines proliferation and reduced tumor burden in an in vivo model of a patient-derived tumor and these encouraging results might be due to *MYC* downregulation [258]. JQ1 also exhibited anti-cancer activity in PCa, especially in CRPC cell lines [183]. It was demonstrated that JQ1 acts downstream of AR, disrupting its recruitment to target gene loci. This compound also has the ability to downregulate either the expression or the oncogenic activity of *MYC* and transmembrane protease serine 2-v-ets avian erythroblastosis virus E26 oncogene homolog (*TMPRSS2-ETS*) gene fusion products. I-CBP112 significantly decreased LNCaP cell proliferation through increased H3K18 acetylation [187]. These data suggest that BET bromodomain inhibitors might be therapeutically useful tools in PCa. However, the molecular mechanisms that determine the activity of BET inhibitors upon MYC and AR regulation in PCa must be further investigated. Two clinical trials with the BET inhibitor OTX015 in solid tumors, including CRPC are ongoing (NCT02698176 and NCT02259114) and might shed some light on the potential clinical usefulness of these compounds.

### Combination strategies: epigenetic modulators and conventional therapy

#### Pre-clinical assays

Co-treatment of DU145 cells with 5-aza-2′-deoxycytidine and sodium butyrate induced site-specific demethylation in the *AR* promoter region with concomitant gene re-expression [259]. In another pre-clinical assay, combination of 5-azacytidine and docetaxel also induced tumor growth delay. In fact, 5-azacytidine sensitized PC-3 and 22Rv1 xenografts to docetaxel, and this combination was not only well tolerated by mice but it was also superior compared to either agent alone [260]. Combined exposure to 5-aza-2′-deoxycytidine and GSK126 (EZH2 inhibitor) showed an additive inhibitory effect on growth of cancer cells in vitro and re-expression of tumor suppressor genes. Moreover, it induced a more powerful in vivo inhibition of PC-3 xenograft tumor growth than 5-aza-2′-deoxycytidine alone [261]. In another study, GSK126 combined with conventional chemotherapy sensitized CRPC cells to apoptosis and growth inhibition both in vitro and in vivo [251]. These results suggest that EZH2 inhibitors might be helpful to increase CRPC patient response to conventional therapy.

PCa cells exposed to vorinostat/SAHA combined with olaparib (a PARP inhibitor) demonstrated a synergistic decrease in cell viability and clonogenicity, as well as an increase in apoptosis and DNA damage compared with single agent, not affecting normal prostate cells [262]. This compound also enhanced radiation-induced apoptosis in DU145 cells [263] and demonstrated a synergistic effect with zoledronic acid, increasing LNCaP and PC-3 cell death [264]. Moreover, low doses of vorinostat/SAHA combined with bicalutamide, synergistically increased apoptosis and decreased cell proliferation [265]. Panobinostat combined with radiotherapy (RT) significantly improved the efficiency of cell death and induced persistent DNA double strand breaks, suggesting that it might increase radiosensitivity of PCa [266]. Moreover, chemosensitivity to gemcitabine was augmented in DU145 cells and xenografts after pre-treatment with low-dose romidepsin [267]. Romidepsin combined with docetaxel not only demonstrated superior cytotoxic effects in CRPC cell lines but it also significantly reduced tumor growth in mice [268]. A combination of sulforaphane, bicalutamide, and enzalutamide enhanced the anti-proliferative effects, decreased tumor cell migration, and reduced PSA and AR expression in LNCaP and C4-2B cells [269].

Anacardic acid sensitized PCa cell lines to radiation therapy by decreasing H2AX and p-H2AX expression [270]. Recently, exposure of enzalutamide-resistant mCRPC cells to BETi (JQ1 and OTX015) resulted in attenuation of AR target genes (*FKBP5*, *KLK3*, *ERG*, and *MYC*) and *AR-v7* expression as well as decreased CRPC cell proliferation in vitro and tumor growth in vivo. Moreover, BETi enhanced the anti-tumor effects of the anti-androgens enzalutamide and ARN509 in a in vivo model [271]. UVI5008, a multi-target epi-drug that inhibits HDACs, Sirtuins, and DNMTs, decreased DU145 cell proliferation, and induced apoptosis by activating initiator and effector caspases and reducing mitochondrial membrane potential [272].

#### Clinical trials

A phase I clinical trial (NCT00503984) with 5-azacytidine combined with docetaxel (alternately escalated in a standard 3 + 3 design) and prednisone (5 mg twice daily continuously), in a 21-day cycle, enrolled 15 mCRPC patients, which had progressed during or within six months of chemotherapy with docetaxel. No dose-limiting toxicity was observed, and the most common adverse event related was neutropenia. A phase II clinical trial-enrolled six patients who received 150 mg/m^2^ of 5-azacytidine for five days followed by 75 mg/m^2^ of docetaxel on day six during 46 cycles. Grade 3 hematologic and non-hematological toxicities were observed, and one patient died from neutropenic sepsis. Subsequently, 5-azacytidine schedule was reduced to 75 mg/m^2^ daily for five days followed by docetaxel. PSA response was observed in 10 of 19 (52.6 %) patients, and the median duration of response was 20.5 weeks. A complete response was achieved in one patient, partial response in two patients, five patients showed stable disease, and two patients experienced disease progression [273].

In a phase I clinical (NCT00565227) which enrolled four CRPC patients, the combination of vorinostat/SAHA, given orally with intravenous docetaxel induced high toxicity, entailing trial closure [274]. A phase I trial (NCT00331955) combined oral vorinostat/SAHA (administered on days 1, 2, and 3 with a planned dose escalation of 600 mg given twice a day in two divided doses) and 20 mg/m^2^ of the topoisomerase II inhibitor doxorubicin (infused on third day, 4 h after the last vorinostat/SAHA dose). Partial response was achieved in one of the two PCa patients enrolled [275]. Sixteen CRPC patients were enrolled in a parallel, two-arm, open-label, phase IA/IB study (NCT00663832), with oral panobinostat alone (20 mg administered on days 1, 3, and 5 for two consecutive weeks) or in combination with docetaxel and prednisone (15 mg of panobinostat administered in the same schedule and 75 mg/m^2^ of docetaxel every 21 days). Partial response was achieved in five (63 %) patients taking the combined therapy whereas none was obtained with panobinostat alone arm. However, patients from both arms showed grade 3 toxicities [276]. A randomized phase I/II trial (NCT00878436) of panobinostat (three different schedules—C1 60 mg/weeek, C2 90 mg/week, C3 120 mg/week, orally) and bicalutamide (50 mg PO daily) was conducted in nine CRPC patients. Grade 3 toxicities were observed and PSA decline ≥50 % was observed in two patients and stable PSA in three patients. As this regimen was well tolerated by the patients showing promising PSA responses, the study proceeded for phase II [277]. A phase I clinical trial (NCT00530907) in which VPA (5.3 mg/kg PO daily) was combined with bevacizumab (11 mg/kg IV once every 14 days) demonstrated that this combination was safe and well tolerated by the patients. One of the six PCa patients (17 %) enrolled in this trial presented stable disease for over 6 months [278].

A phase II clinical trial evaluated the combinatory effect of curcumin, prednisone, and docetaxel in 30 CRPC patients. Docetaxel and prednisone were administered in standard conditions for six cycles and curcumin at 6000 mg/day (day −4 to day +2 of docetaxel). This schedule was well tolerated by the patients, with no significant toxicities observed. Decreased PSA levels were observed in 59 % of patients, and 40 % of evaluable patients presented a partial response [279]. Another clinical trial (NCT01917890) investigated the efficacy of curcumin and radiotherapy. PCa patients (*n* = 40) undergoing external beam radiotherapy were randomly selected to receive 3 g/day curcumin orally (*n* = 20) or a placebo (*n* = 20). Patients who received curcumin present reduced urinary symptoms related to radiotherapy, suggesting that this compound could offer radioprotective effects [280].

## Conclusion and future directions

Considering the success of epigenetic drugs in acute leukemia and myelodysplastic syndrome, there is a growing interest for their use in solid tumors. The results of epigenetic-based therapy in cutaneous lymphomas further suggest the possibility that solid tumors may also respond to such treatment.

Concerning DNMT inhibitors, the lack of success of azanucleosides observed in solid tumors, including PCa, might be due to the fact that they are mostly effective in highly proliferative tumors and the rate of active cell division is much lower in solid tumors, compared to hematolymphoid neoplasms. Moreover, the potential of demethylating agents to cause global hypomethylation leading to unwanted activation of imprinted or silenced genes is an additional concern. Therefore, their lack of specificity might paradoxically contribute to tumorigenesis and increased disease aggressiveness due to upregulation of genes involved in metastasis. Indeed, several studies have shown incredible substantial decrease in m^5^C content alongside with specific demethylation of tumor suppressor gene promoters with concomitant re-expression [281]. Treatment with azanucleosides is also associated with hematopoietic, nervous, and metabolic toxicity. However, they usually display a lower toxicity profile than conventional chemotherapy. Although, non-nucleoside inhibitor compounds are less cytotoxic than nucleoside inhibitors, they proved to be less effective than azanucleosides at inhibiting DNA methylation and reactivating gene expression [69, 102, 116].

Considering histone modulators, the best studied thus far are HDACi. However, these compounds are not specific and they rather act on non-histone proteins in addition to histones, which could contribute to more aggressive side effects. Nevertheless, it was shown that these drugs preferentially target genes that have become abnormally silenced in cancer and, indeed, the chromatin silencing structure induced by cancer is more susceptible to reactivation than the structure of physiologically compacted chromatin [282]. The ideal treatment would be the one that could selectively reverse hypermethylation of tumor suppressor genes’ promoters, reestablishing its function, without causing global demethylation of the genome. Eventually, the combination of DNMTi with HDACi and conventional chemotherapy might be a promising strategy for the treatment of PCa patients. Nevertheless, additional studies are required to assess the role of DNMTi, especially non-nucleoside analogues, as therapeutic options for PCa.

Of some concern, much of the clinical evaluation of epigenetic therapeutics in PCa to date has been undertaken in late stage, heavily pre-treated mCRPC patients, commonly without a patient stratification strategy and with agents of sometimes poorly defined specificity for epigenetic effect (particularly for “repurposed” drugs). Since epigenetics is a complex process of gene regulation, there is a need for evaluation of agents where we understand clearly the epigenetic target(s), in clinical trials where we also test potential predictive biomarkers to select patients that would benefit from these therapies. Ideally, pre-clinical studies should focus on providing patient stratification hypotheses that we can take through to the clinic. Earlier stage disease, for example, patients who have biochemical recurrence after radical prostatectomy or patients receiving ADT prior to transition to a CRPC phenotype might represent more relevant clinical settings for assessment of epigenetic therapeutics [28]. It might also be useful to evaluate other parameters. For example, low doses of 5-aza-2′-deoxycytidine have shown to be able to minimize toxicity while potentially improving the targeted effects of DNA hypomethylation [283]. Thus, the hypothesis of reducing dose to an epigenetic but not cytotoxic level might allow us to target better the therapeutic index between efficacy and safety, particularly in combinations of either epigenetic/epigenetic and epigenetic/non-epigenetic drugs. In addition, we have relatively limited experience of the clinical impact of prolonged maintenance treatment with epigenetic agents, at high or low dose, in terms of toxicity profiles or mechanisms of emergent acquired resistance to therapy. Together with increased insight into the molecular mechanisms underlying the activity of epigenetic-based drugs, linking the rapidly advancing biological understanding of the disease for more precise selection of PCa subtypes for clinical trials will hopefully foster successful clinical validation of these drugs for the treatment of PCa.

## Abbreviations

*ABCB1*: ATP binding cassette subfamily B member 1
AdoHcy: *S*-Adenosyl-l-homocysteine
AdoMet: *S*-Adenosyl-methionine
ADT: Androgen deprivation therapy
ALDH1: Aldehyde dehydrogenase 1
ALL: Acute lymphocytic leukemia
AML: Acute myeloid leukemia
*APC*: Adenomatous polyposis coli
AR: Androgen receptor
*BCL2*: B cell CLL/lymphoma 2
BET: Bromodomain and extraterminal domain family
BRD: Bromodomain-containing protein
*CCND2*: Cyclin D2
*COX2*: Cyclooxygenase-2
CRPC: Castration-resistant prostate cancer
CTC: Circulating tumor cells
DHT: Dihydrotestosterone
DNMT: DNA methyltransferase
DNMTi: DNA methyltransferases inhibitors
DZNeP: 3-Dezaneplanocin-A
*EFEMP*: EGF-containing fibulin-like extracellular matrix protein
EGCG: Epigallocatechin-3-gallate
EGF: Epidermal growth factor
EGFR: Epidermal growth factor receptor
EMT: Epithelial mesenchymal transition
ERβ: Estrogen receptor β
*ETS*: v-ets Avian erythroblastosis virus E26 oncogene homolog
EZH2: Enhancer of zeste 2 polycomb repressive complex 2 subunit
FAD: Flavin adenine dinucleotide
FDA: Food and Drug Administration
GnRH: Gonadotropin-realizing hormone
*GSTP1*: Glutathione S-transferase pi 1
HAT: Histone acetyltransferase
HATi: Histone acetyltransferase inhibitors
HDAC: Histone deacetylases
HDACi: Histone deacetylase inhibitors
HDM: Histone demethylase
HDMi: Histone demethylase inhibitor
HER2: Human epidermal growth factor receptor 2
HMT: Histone methyltransferase
HMTi: Histone methyltransferase inhibitor
IL-6: Interleukin 6
*IGF2*: Insulin-like growth factor 2
I.v.: Intravenous
KDM1A: Lysine-specific demethylase 1A
LSD1: Lysine (K)-specific demethylase 1A
MAO: Monoamine oxidase
mCRPC: Metastatic castration-resistant prostate cancer
*MeCP2*: Methyl CpG binding protein 2
MDS: Myelodysplastic Syndrome
*MGMT*: *O*-6-Methylguanine-DNA methyltransferase
*MYC*: v-Myc avian myelocytomatosis viral oncogene homolog
*NDRG1*: N-Myc downstream regulated gene-1
*Neurog1*: Neurogenin 1
NF-kB: Nuclear factor kappa B
PCa: Prostate cancer
PCAF: p300/CBP associated factor
PEITC: Phenethyl isothiocyanate
PFS: Progression-free survival
PIN: Prostatic intraepithelial neoplasia
*PLAU*: Urokinase plasminogen activator
PRC2: Polycomb repressive complex 2
PSA: Prostate-specific antigen
PSA-DT: PSA doubling time
*PTGS2*: Prostaglandin-endoperoxide synthase 2
*PTMs*: Histone post-translational modifications
*RARβ2*: Retinoic acid receptor beta 2
*RASSF1A*: Ras association domain family protein 1, isoform A
RT: Radiotherapy
SAHA: Suberoylanilide hydroxamic acid
SAM: *S*-Adenosylmethionine
SIRT: Sirtuin
*SPARC*: Secreted protein acid and cysteine rich
TIMP: Tissue Inhibitor of Metalloproteinase
*TMPRSS2*: Transmembrane protease serine 2
TSA: Trichostatin A
UTR: Untranslated region
VPA: Valproic acid
